# A Rare Case of Gastric Phytobezoar (Diospyrobezoar) in a Healthy Adult

**DOI:** 10.7759/cureus.68353

**Published:** 2024-08-31

**Authors:** Ahmed M Odeh, Ahmed A Alkhalifa, Mohammed A AlHajji, Alwayah J Alahmed, Jawad H Alsalman, Abdulrahman M AlMulhem, Mohammed S AlGhadeer, Ezzeddin Kurdi, Fatima S Albader, Abdulmohsen Alsuwaigh, Mohammad A Khan

**Affiliations:** 1 General and Laparoscopic Surgery, Al Ahsa Health Cluster, Al Ahsa, SAU; 2 General Surgery, Al Ahsa Health Cluster, Al Ahsa, SAU; 3 Radiology, Diagnostic Neuroradiology, Al Ahsa Health Cluster, Al Ahsa, SAU; 4 Internal Medicine, Al Ahsa Health Cluster, Al Ahsa, SAU; 5 Nursing, Al Ahsa Health Cluster, Al Ahsa, SAU; 6 General Surgery, Bariatric and Laparoscopy, Al Ahsa Health Cluster, Al Ahsa, SAU; 7 General Surgery, Max Super Speciality Hospital, Dehradun, IND

**Keywords:** laparoscopic extraction, persimmons, diospyrobezoar, gastric foreign body, phytobezoar

## Abstract

Phytobezoars are solid masses of indigestible plant material and are a common type of gastrointestinal bezoar, with varying incidences globally. These bezoars typically form from the ingestion of high-fiber fruits and vegetables and are associated with factors such as decreased gastric acid production and delayed gastric emptying. We present a case of a 35-year-old healthy man with recurrent upper abdominal pain, nausea, a rolling ball sensation in the abdominal region, and a history of consuming unripe persimmons. Imaging revealed the presence of phytobezoars in the stomach, leading to unsuccessful endoscopic attempts at removal. Laparoscopic extraction was eventually performed successfully after failed conservative management. The case highlights the rarity of diospyrobezoars, a subtype of phytobezoars formed from persimmon ingestion, and the challenges in their management. Surgical intervention, particularly laparoscopic extraction, can be effective but carries risks such as surgical site infections. Comprehensive care involving diagnostic imaging, non-surgical interventions, and surgical techniques is crucial for the successful management of phytobezoars. Phytobezoars, though relatively common, present unique diagnostic and management challenges, especially when formed from specific dietary factors such as persimmons. Understanding their epidemiology, clinical manifestations, and treatment options, including the role of laparoscopic surgery, is essential for optimizing patient outcomes and minimizing complications such as surgical site infections.

## Introduction

Phytobezoars are solid masses composed of indigestible plant material that accumulate in the gastrointestinal tract, most commonly in the stomach [1,2]. These bezoars are one of the most prevalent types of gastrointestinal bezoars, with a reported incidence ranging from 0.4% to 4% [3].

Phytobezoars typically form from the ingestion of certain fruits and vegetables that are high in cellulose, hemicellulose, and lignin, such as persimmons, pumpkins, prunes, and raisins [1,2]. Patients with decreased gastric acid production, delayed gastric emptying, or a history of gastric surgery are at an increased risk of developing phytobezoars [2,3].

The epidemiology of phytobezoars varies across regions and populations. In Western countries, they are more common in older adults with a history of gastrointestinal surgeries, while in certain Asian and Middle Eastern countries, they are more prevalent in younger individuals consuming large quantities of persimmons [1-3]. Understanding the epidemiology and risk factors is crucial for timely diagnosis and management of this condition.

## Case presentation

A 35-year-old man, not known to have any chronic medical illness, presented to our clinic with multiple attacks of upper abdominal pain, dull and vague in nature, associated with nausea and early satiety. The patient reports experiencing a sensation of a ball moving around in his stomach, particularly noticeable when he shifts positions during sleep. The patient provided a history of consuming a large quantity of unripe persimmons, which preceded the onset of the current symptoms.

**Figure 1:**
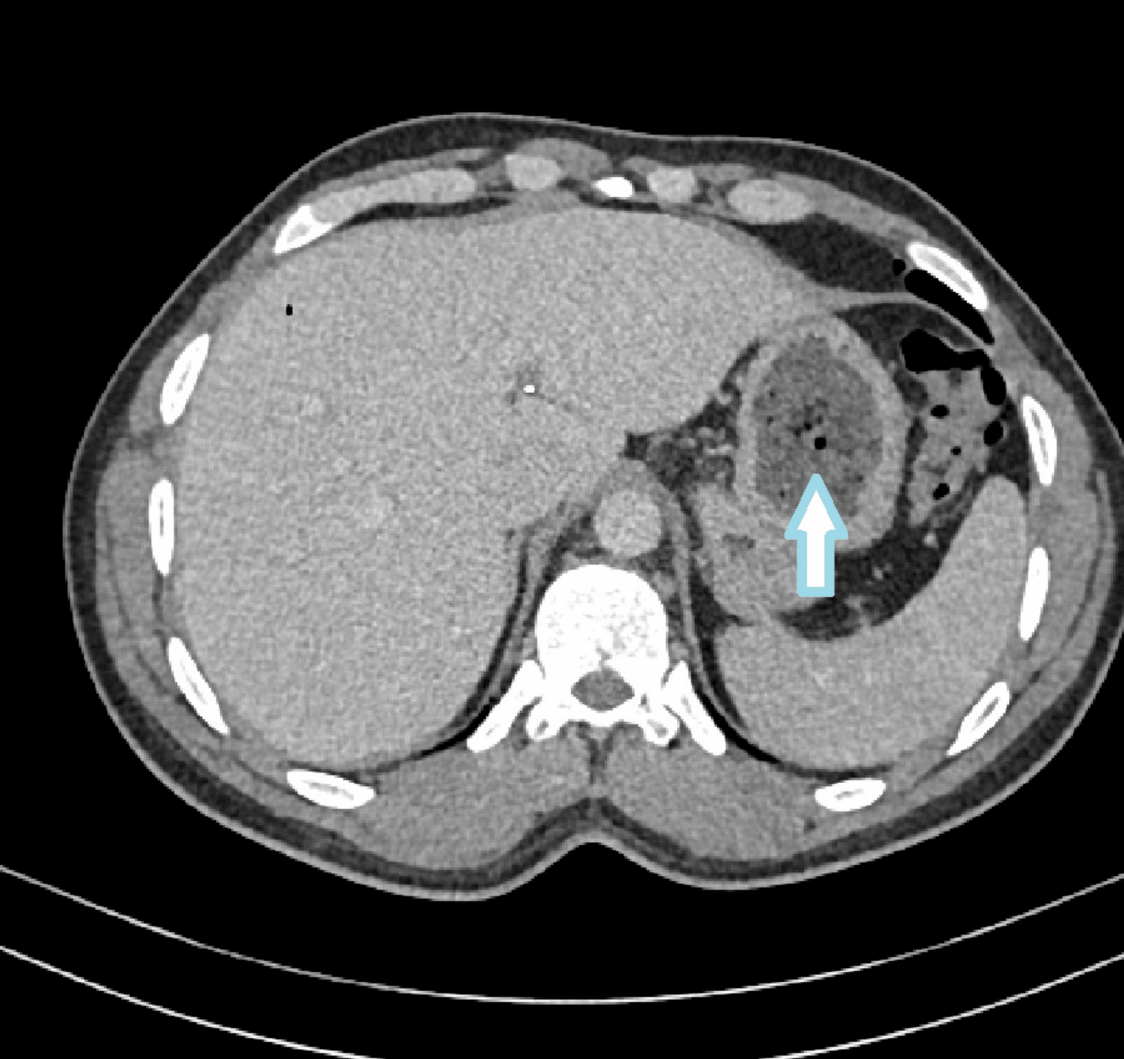
The stomach shows two inhomogeneous intraluminal masses with a mottled gas pattern located in the body and antrum, measuring about 6 x 5 cm and 5 x 5 cm in their maximum dimension, respectively (axial view)

**Figure 2:**
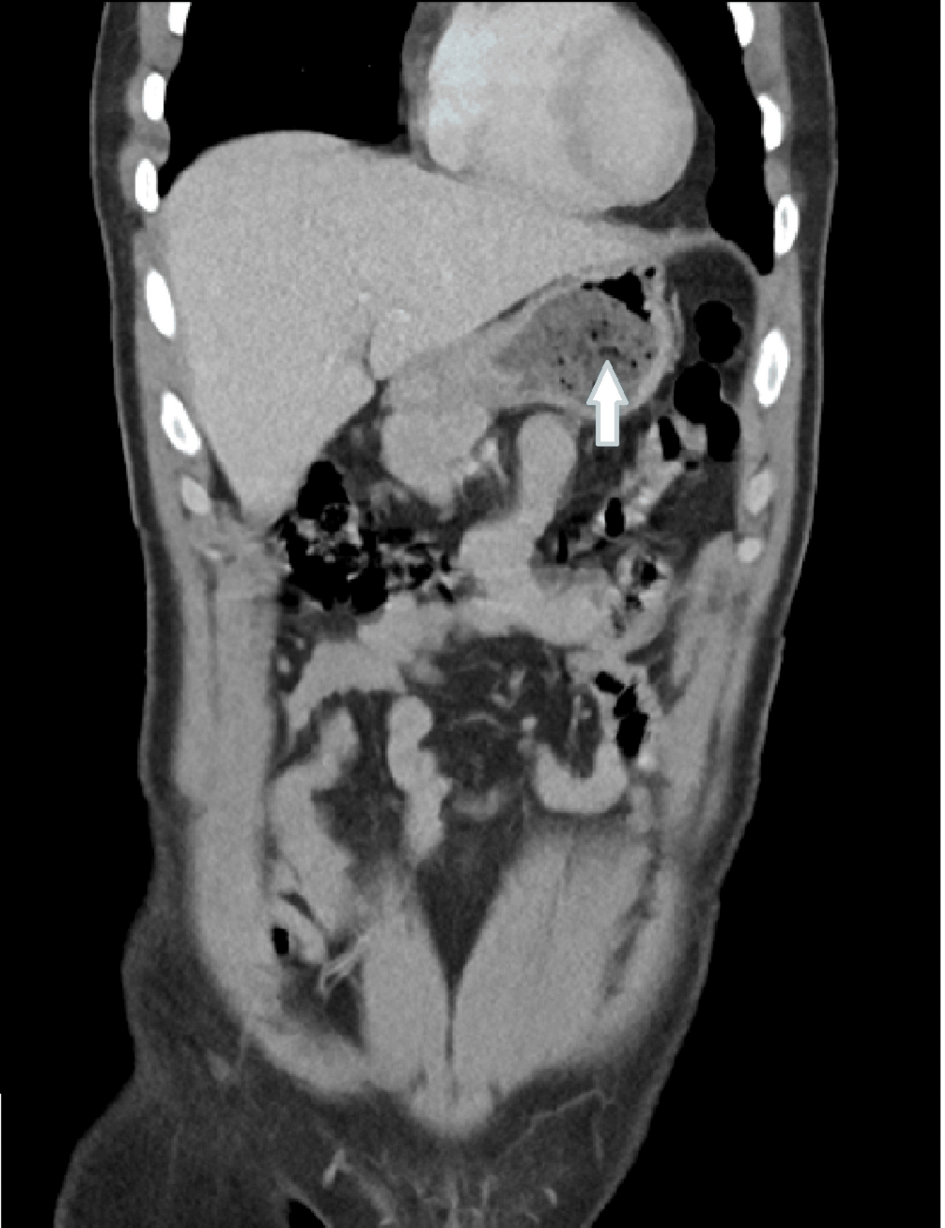
The stomach shows two inhomogeneous intraluminal masses with a mottled gas pattern located in the the body and antrum, measuring about 6 x 5 cm and 5 x 5 cm in their maximum dimension, respectively (coronal view)

**Figure 3:**
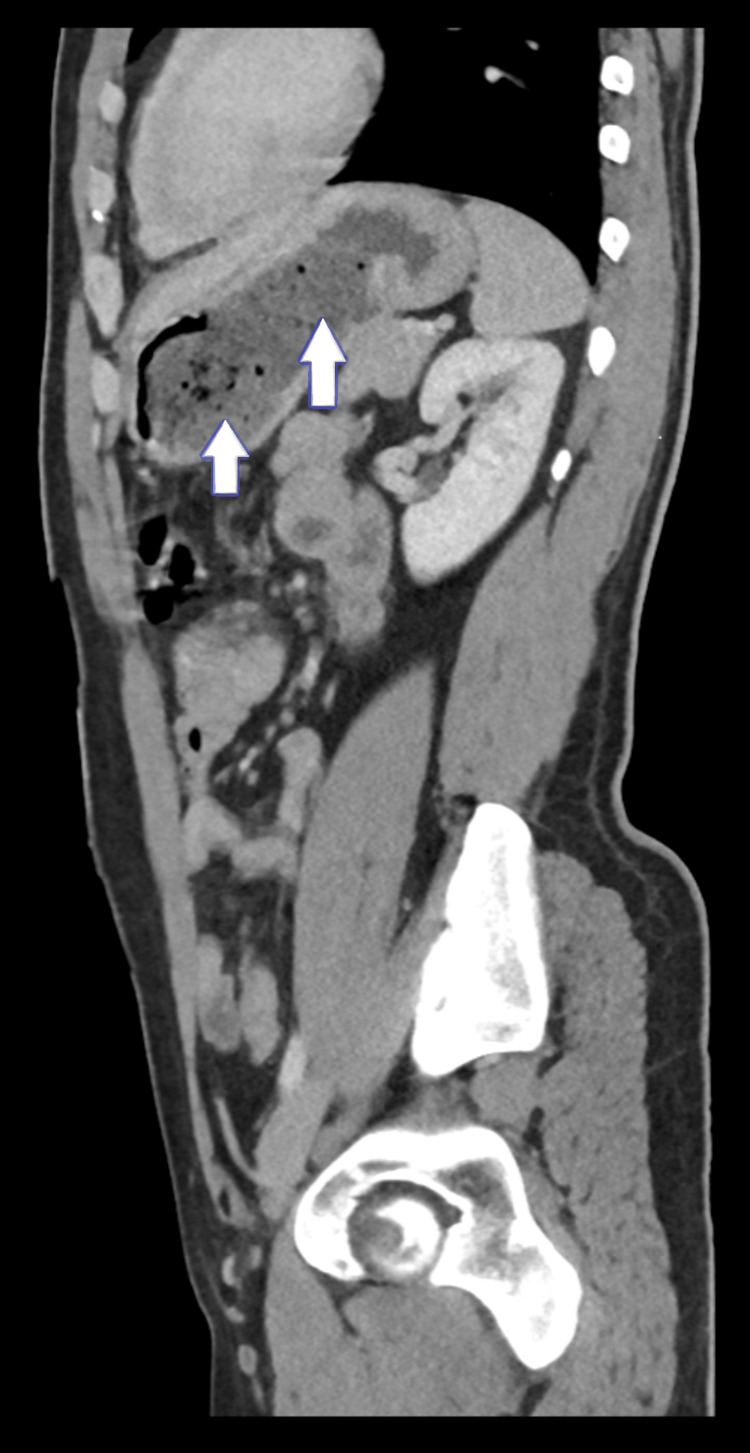
The stomach shows two inhomogeneous intraluminal masses with a mottled gas pattern located in the body and antrum, measuring about 6 x 5 cm and 5 x 5 cm in their maximum dimension, respectively (sagittal view)

Upon general examination, the patient appears to be in overall good health. However, the abdominal examination reveals an ill-defined palpable fullness in the epigastric region, which is mildly tender upon palpation. Comprehensive blood investigations, including a complete blood count and biochemistry tests, were conducted for the patient, with all results showing no significant abnormalities or deviations from normal ranges( Table 1). A CT scan of the abdomen and pelvis revealed the presence of two inhomogeneous intraluminal masses with a mottled gas pattern, situated within the body and antrum of the stomach. These masses measure approximately 6x5 cm and 5x5 cm in their maximal dimensions, findings that are consistent with the presence of phytobezoars (Figures 1-3).

**Table 1:** Lab results

WBC	6.60	BUN	2.00	ALT	34
HGB	15.6	Creatinine	63.0	AST	25
PLT	216	Sodium (NA)	139	Alkaline Phosphate	93
		Potassium (K)	4.19	Total Bilirubin	7

Gastroenterology consultation was sought, and the patient underwent two attempts at endoscopic procedures. Unfortunately, both attempts were unsuccessful due to the difficulty in fragmenting the phytobezoar using an endoscopic snare.

**Figure 4:**
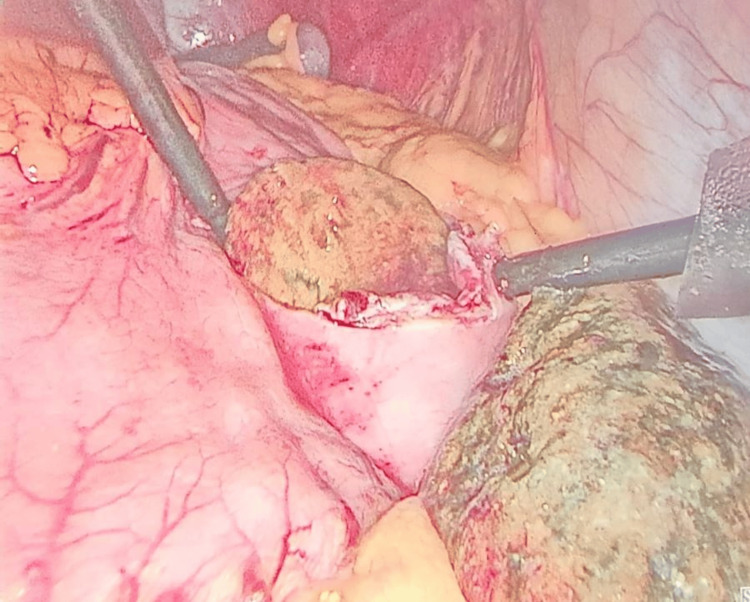
Intaroperative view of the laparoscopic extraction of the phytobezoar

**Figure 5:**
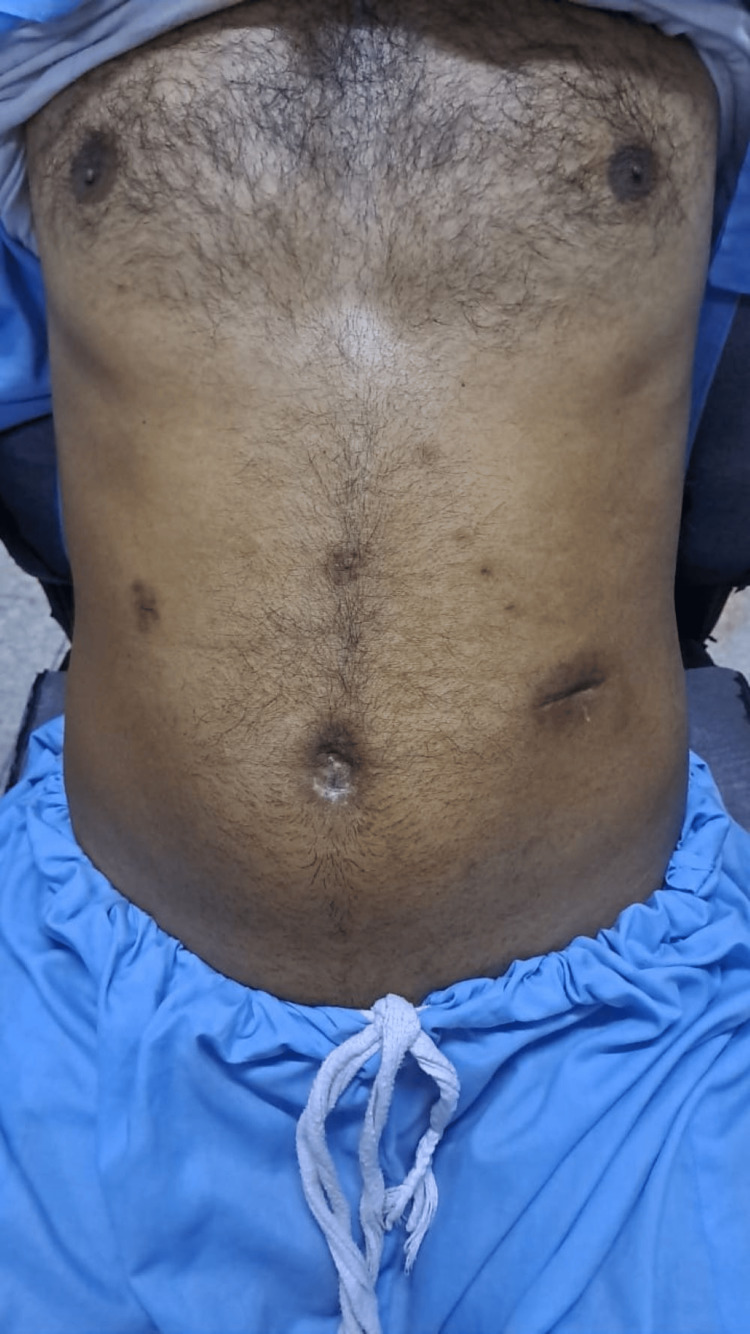
Post-operative follow-up picture showing the healed wound at the operative site

A trial of conservative management was implemented, involving instructions for the patient to consume increased quantities of beverages. However, this approach did not yield any benefits. It was decided to proceed with laparoscopic extraction of the phytobezoar, and arrangements were made for the patient to undergo surgery. The procedure was successfully performed, resulting in the extraction of the phytobezoar (Figure 4). The patient subsequently had an uneventful postoperative recovery (Figure 5).

## Discussion

The case describes a 35-year-old man presenting with symptoms suggestive of a gastric bezoar, specifically a phytobezoar. The patient reported a history of consuming a large quantity of unripe persimmons, which is a common dietary factor associated with the development of phytobezoars.

Diospyrobezoars, a specific subtype of phytobezoars formed from the ingestion of persimmons, are relatively rare compared to other types of bezoars. The formation of diospyrobezoars from the ingestion of unripe persimmons can be attributed to the unique composition of the fruit. Persimmons are rich in fiber and calories, with their pulp primarily composed of mucilage and pectin substances. These components give the fruit its characteristic appearance [3,4].

One key element found in persimmons is a high concentration of tannin, specifically shibuol. When this tannin comes into contact with gastric juice in the stomach, it can precipitate. In the acidic environment of the stomach, the tannin undergoes polymerization, forming a coagulum that includes cellulose, hemicellulose, and protein. This coagulum serves as the foundation of the bezoar. As this cluster continues to evolve, it undergoes dehydration processes. The kinetic energy provided by the movements of the gastric wall contributes to the consolidation of the bezoar, making it more solid and compact. This process ultimately leads to the formation of the diospyrobezoar, a unique type of phytobezoar with distinct properties and challenges in diagnosis and management [5,6].

The patient's symptoms, including upper abdominal pain, nausea, early satiety, and a sensation of a "ball moving around" in the stomach, are consistent with the clinical manifestations of bezoars reported in the literature [1,2,4,6-8]. The workup included comprehensive blood tests, which were unremarkable, and a CT scan of the abdomen and pelvis, which revealed the characteristic findings of phytobezoars in the stomach [1,2,4,5-8].

The management of bezoars often involves a combination of non-surgical (dissolving agents, Coca-Cola, endoscopic fragmentation, and extraction) and surgical approaches [4,7-9]. In this case, the patient failed the non-surgical approach and underwent two unsuccessful attempts at endoscopic fragmentation and removal of the phytobezoars, as reported in some of the references [4,5-9]. When non-surgical options are not feasible, surgical intervention may be necessary.

The use of laparoscopic techniques for the removal of bezoars can be a minimally invasive alternative to open surgery [1]. In our case, the patient underwent a successful laparoscopic extraction of the phytobezoar, which aligns with the advantages of the laparoscopic approach reported in the literature, such as reduced postoperative pain and shorter hospital stay as compared with conventional open laparotomy [1].

However, surgical procedures, including laparoscopic approaches, carry inherent risks. A study by Kement et al. reported a surgical site infection rate of 10.5% in patients undergoing laparoscopic removal of bezoars [6]. Additionally, a study by Iwamuro et al. found that the overall incidence of postoperative complications, including surgical site infections, in patients undergoing surgical treatment for bezoars was 19.5% [4,6]. In our case, we encountered a superficial surgical site infection, which was treated by opening a few stitches and draining the small collection, allowing healing by secondary intention, along with the administration of antibiotics. The patient did well and was reviewed in the outpatient department in good condition.

The risk of surgical site infection can be influenced by various factors, such as the patient's underlying health status, the complexity of the surgical procedure, and the presence of contamination during the operation [9,6]. Appropriate antibiotic prophylaxis, meticulous surgical technique, and postoperative wound care are crucial in reducing the risk of surgical site infections in these cases [8,9].

## Conclusions

This case highlights the importance of a comprehensive approach to the diagnosis and management of bezoars, which can present with a variety of gastrointestinal symptoms even in healthy individuals. The literature provides valuable insights into the different types of bezoars, their clinical manifestations, potential complications, and the role of both non-surgical and surgical interventions, including the emerging use of laparoscopic techniques. The successful laparoscopic extraction of the phytobezoar, in this case, is consistent with the findings reported in the literature, but it is crucial to be aware of the potential risk of surgical site infections as a complication of the surgical management of bezoars.
